# FlyBase 102—advanced approaches to interrogating FlyBase

**DOI:** 10.1093/nar/gkt1092

**Published:** 2013-11-13

**Authors:** Susan E. St. Pierre, Laura Ponting, Raymund Stefancsik, Peter McQuilton, the FlyBase Consortium

**Affiliations:** ^1^The Biological Laboratories, Harvard University, 16 Divinity Avenue, Cambridge, MA 02138, USA and ^2^Department of Genetics, University of Cambridge, Downing Street, Cambridge CB2 3EH, UK

## Abstract

FlyBase (http://flybase.org) is the leading website and database of *Drosophila* genes and genomes. Whether you are using the fruit fly *Drosophila melanogaster* as an experimental system or wish to understand *Drosophila* biological knowledge in relation to human disease or to other model systems, FlyBase can help you successfully find the information you are looking for. Here, we demonstrate some of our more advanced searching systems and highlight some of our new tools for searching the wealth of data on FlyBase. The first section explores gene function in FlyBase, using our TermLink tool to search with Controlled Vocabulary terms and our new RNA-Seq Search tool to search gene expression. The second section of this article describes a few ways to search genomic data in FlyBase, using our BLAST server and the new implementation of GBrowse 2, as well as our new FeatureMapper tool. Finally, we move on to discuss our most powerful search tool, QueryBuilder, before describing pre-computed cuts of the data and how to query the database programmatically.

## INTRODUCTION

### What is FlyBase?

FlyBase (http://flybase.org), a database of *Drosophila* genes and genomes, was created in 1992 as a resource for collecting and disseminating *Drosophila*-related information. Over 20 years later the website contains over 2.5 million report pages incorporating data from over 42 000 primary *Drosophila* research papers and an ever-increasing number of genome-scale projects. As the amount, detail and scope of data in FlyBase has increased, we have expanded and improved the range of data-retrieval tools to ensure that the data in FlyBase are as accessible as possible.

### Data in FlyBase

FlyBase curates a variety of data from published biological literature, including phenotype, gene expression, interactions (genetic and physical), gene ontology (GO) information (1) and many others. These data are organized in ∼31 different data-type reports such as the Gene Report or the Allele Report. The range of data we provide increases and changes as new types of data become available. For example, most recently, we have improved the ortholog data we provide in collaboration with OrthoDB (2) and have added the modENCODE RNA-Seq data (3).

### Different users have different needs

A diverse range of researchers access FlyBase, and many are coming from other fields to find information about the fly ortholog of their gene of interest. To cater to such a wide array of different users we provide a variety of search and data-mining tools. The tools we provide range from straightforward general search tools and data-type-specific tools to more sophisticated search tools that facilitate wide-range querying of multiple data types simultaneously.

For those wishing to carry out straightforward searches, the FlyBase homepage contains the search tool QuickSearch, as well as quick links, via large icons, to a variety of other popular tools, such as BLAST (4) and GBrowse (5). All tools in FlyBase can be found by using the Tools drop-down menu in our navigation bar, found on every FlyBase page. More detailed information about how to navigate the homepage and how to use QuickSearch and GBrowse are covered in a previous paper (6).

In this article, we will focus on some of the more advanced search tools in FlyBase and will also highlight several new and revamped tools. We will do this by providing step-by-step examples of complex queries to highlight key features. We hope to demonstrate the utility of our existing tools as well as to provide users with insights into how to mine the rich data in FlyBase more thoroughly and deeply. All searches described herein have been carried out using the FB2013_05 release of FlyBase.

## EXPLORING GENE FUNCTION

### Performing and refining Controlled Vocabulary searches using TermLink and Batch Download

A Controlled Vocabulary (CV) is a standardized set of descriptive terms used to categorize and annotate data captured from papers. TermLink (http://flybase.org/static_pages/termlink/termlink.html) allows focused searches of FlyBase data using CVs. Prior knowledge of the precise terms used is not necessary. Abbreviations (e.g. ‘CNS’, ‘VNC’), plurals, alternative spellings (e.g. ‘oesophagus’) and alternative terms are recorded as synonyms of the valid CV terms; a TermLink query will automatically search all synonyms. In addition, CV terms are organized into hierarchies that allow the grouping of terms, and searches take these hierarchies into account; for example, ‘wing vein’ is below ‘wing’ in the hierarchy (a child term) so searching ‘wing’ will return all alleles that have been annotated with either term (see our video tutorial of TermLink here: http://youtu.be/XNuYMZzEtCU). Examples of the CVs available on FlyBase include the GOs (molecular function, biological process and cellular component) and sequence ontology, as well as FlyBase-developed ontologies such as anatomy (e.g. adult head), developmental stage (e.g. pre-pupal stage) and phenotypic class (e.g. lethal, circadian rhythm defective).

#### Example

Here, we will use TermLink to identify those genes with mutant alleles that produce an abnormal stem cell phenotype. To illustrate the utility of this tool for an audience beyond the *Drosophila* research community, we will then use the list of identified genes to find their human orthologs.

**Figure 1.**
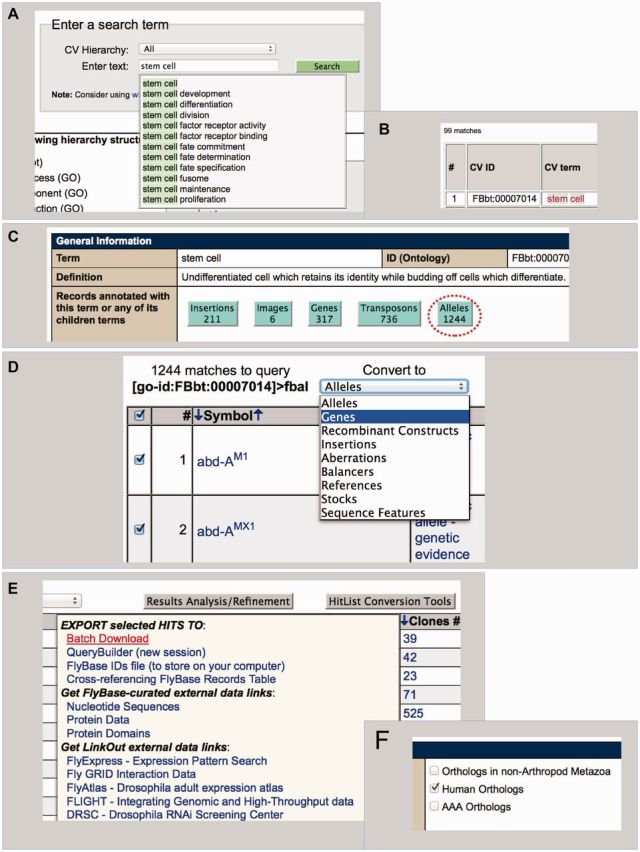
TermLink search example. **(A)** Search for ‘stem cell’ in TermLink. **(B)** Select ‘stem cell’ from the FBbt ontology. **(C)** Click ‘Alleles’ (red dashed circle) to see alleles showing stem-cell-related phenotype. **(D)** On the resulting hitlist page, select ‘Genes’ (highlighted) from the ‘Convert to’ drop-down menu. **(E)** Export the list of genes to ‘Batch Download’ (in red and underlined) using the ‘HitList Conversion Tools’ menu. **(F)** Using the ‘Select fields’ on the batch download page, select the ‘Human Orthologs’ radio button.

The ‘TermLink Search Page’ contains two options—a search box that allows searching of all the CV hierarchies and a CV tree navigation tool for browsing the FlyBase CV hierarchy structures. For our example, we will search for ‘stem cell’ in TermLink (Figure 1A). The search returns a page with CV terms that include the string ‘stem cell’. After clicking on the term ‘stem cell’ (FBbt:00007014) (Figure 1B), we are presented with the CV term report that contains the definition of the chosen term ‘stem cell’ (Figure 1C). Below the definition is a set of buttons indicating the number of FlyBase pages where this term (or any of its child terms) has been annotated (Figure 1C). Please note that CV terms are annotated to different data types for different reasons. Genes are annotated with ‘stem cell’ based on expression data, while alleles are annotated with ‘stem cell’ based on phenotypes. Hence, clicking on the ‘Genes’ button would return a hitlist of genes that FlyBase reports as expressed in stem cells. For our example, we are interested in phenotypes so we click on the ‘Alleles’ button (Figure 1C, dashed circle), which reveals a hitlist of all the alleles annotated with the ‘stem cell’ anatomy term, indicating that these alleles exhibit a phenotype manifested in the stem cells.

The ‘Convert to’ drop-down menu on the page allows for the conversion of the current hitlist (in this case, alleles) into a hitlist of related genes, recombinant constructs, insertions, aberrations, balancers, references, stocks or sequence features. To obtain the list of genes that are involved in stem cell-related abnormalities, we choose the ‘Convert to-Genes’ option, resulting in 746 matches (Figure 1D).

For our list of genes, we would like a corresponding list of human orthologs, and the Batch Download tool can be used to quickly obtain this list. In brief, Batch Download (http://flybase.org/static_pages/downloads/ID.html) is designed to allow simple access to a wide variety of data for a specified list of genes, alleles, insertions, etc. Most commonly, it is used to obtain the contents of a certain data field or fields for a large number of objects at once. Our hitlist of genes can be exported directly to Batch Download using the ‘HitList Conversion Tools’ button at the top right corner of the hitlist and selecting ‘Batch Download’ from the list of options (Figure 1E, note that there are several different export options from a hitlist, both internal and external to FlyBase). This brings up a ‘Batch Download’ input page in which the IDs for our genes of interest have been inserted. The default behavior for Batch download is to have the output data displayed in a browser window as an HTML table (see our help documentation for how to have results sent to a text file). Clicking ‘Select Fields’ brings us to a page that looks similar to the Gene Report in layout. We may now select one or more fields for download. For our ortholog analysis, we scroll down the page to the ‘Orthologs’ section and choose the ‘Human Orthologs’ option (Figure 1F); other options may be added if needed (for most Batch Download lists, selection of ‘Symbol’ is advised). After clicking the button ‘Get Field Data’ (on the top right-hand side of the page), we obtain a table of our genes along with their respective human orthologs. If we instead batch download as a tab separated file and open as a spreadsheet, we can rapidly determine that 513 of the 746 fly genes implicated in stem cell biology have at least one identified human ortholog.

### RNA-Seq Search with query analysis

RNA-Seq coverage data (3) are aligned to the genome and are viewable on our genome browser, GBrowse. To enable gene-based querying of RNA-Seq coverage data, FlyBase employed a method of quantifying the expression across the transcribed region of each gene and assigning a value equal to the sum of all read counts over the uniquely transcribed region; these values are called reads per kilobase per million reads (RPKM) (7). The ‘uniquely transcribed’ region is based upon our gene model annotations and is important in cases of overlapping or nested genes (see *CG42358* and *CG42359*, or *nos* and *CG11779*). Only the non-overlapping portions of each gene are considered when making RPKM calculations (8). For the purposes of displaying patterns of expression on gene reports and also for searching, the RPKM values were assigned to one of eight different bins ranging from ‘no expression’ to ‘extremely high expression’. For several different RNA-Seq datasets, these bin values are displayed on Gene Reports as histograms to illustrate the pattern of expression either through developmental time, across different tissues, in different cell lines or in response to different treatments (3).

RPKM values have also been incorporated into a combinatorial query interface for searching expression patterns of all annotated genes, called RNA-Seq Search. This tool is available both from our home page icon bar as well as from the Tools drop-down menu.

#### Example

To illustrate RNA-Seq Search’s utility, we will attempt to find potential maternal effect genes that have not yet been described as such in FlyBase. Maternal effect gene products are usually highly abundant early in embryogenesis due to maternal loading of mRNAs and proteins. They are also typically highly expressed in the ovary.

**Figure 2.**
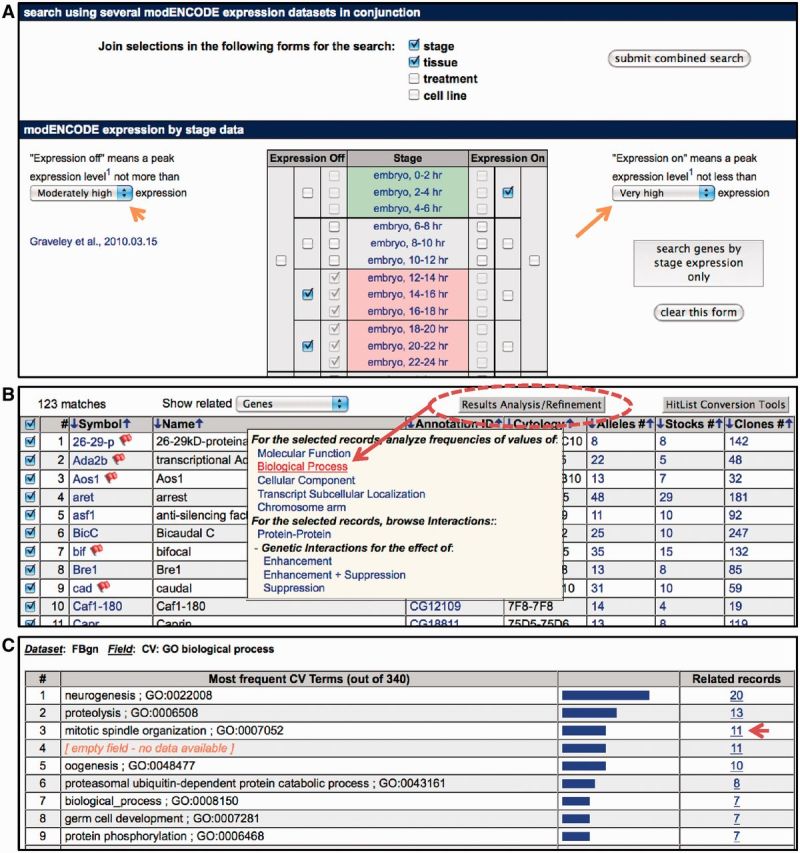
RNA-Seq Search and Results Analysis/Refinement. **(A)** Portion of input page for RNA-Seq Search. Please note that the screenshot is cut off, and the section of the search for ‘tissue’ is not shown. Orange arrowhead on left indicates the drop-down menu to set the level of ‘Expression off’, and orange arrow on right indicates the same for ‘Expression on’. **(B)** Top of hitlist for search results. Red dotted circle indicates Results Analysis/Refinement button, and inset panel shows the options. Red arrow points to ‘Biological Process’ which is selected. Results are shown in **(C)**. Red arrowhead indicates number of records annotated with the term ‘mitotic spindle organization’.

To use RNA-Seq Search, we first need to select the expression datasets to search. One or more datasets can be searched at the same time. In this example, we will use the ‘stage’ and ‘tissue’ datasets (see Figure 2A). First, we will look for genes with expression levels of ‘very high’ or more in early embryos (0–6 h AEL) by selecting ‘very high’ as the ‘Expression on’ level and ticking the early embryo boxes. To eliminate genes that are simply very highly expressed throughout development, we can look for genes with lower expression later in embryonic development. To do this, we adjust the definition of ‘expression off’ to ‘moderately high’ and tick the boxes next to the later embryonic stages (see Figure 2A). Second, we will move down to the ‘tissue’ data set and after setting the levels for ‘Expression on’ and ‘Expression off’, we can tick the boxes corresponding to ‘ovary’ (not shown in Figure 2). In summary, we are looking for genes with very high expression in early embryos and ovaries, but with no more than moderately high expression in later embryonic stages. Running this search gives 123 matches.

Many of these hits are to known maternal effect genes such as *oskar*, *nanos* and *grapes*. To filter out these known results, we could export our list to QueryBuilder (using the ‘Hitlist Conversion Tools’ button at the top right of the hitlist) and exclude genes with the phrase ‘maternal effect’ found on the gene report (please see following sections for description of how to use QueryBuilder). Doing so would leave us with 89 matches, many of which are unnamed genes, suggesting that their function has not been characterized.

Alternatively, our list of 123 genes could be evaluated further using our hitlist Results Analysis/Refinement options (see Figure 2B). For our hitlist of genes, we could analyze the frequency of annotated GO terms, or we could browse for reported genetic or physical interactors. We can also refine our query. To limit our hitlist to only those genes with a known role in, for example, mitotic spindle organization, we choose ‘Biological Process’ from the drop-down menu (Figure 2B, arrow). This generates a list of Biological Process CV terms ordered by frequency (Figure 2C). From this list we can select the records annotated with the term ‘mitotic spindle organization’ (Figure 2C, arrowhead) which will generate a new refined hitlist.

In summary, the breadth of our high-throughput expression data enables one to ask many different questions. When combined with our literature-curated data through the use of our search tools, the expression data can help researchers begin to address complex biological questions.

## EXPLORING THE GENOME

### BLAST and GBrowse2

FlyBase offers a stand-alone BLAST server for 49 different arthropod genomes (http://flybase.org/blast/). These range from 26 Dipteran species to *Acromyrmex echinatior* (Panamanian leafcutter ant) and *Rhipicephalus microplus* (Southern cattle tick). On the BLAST query page, the species are arranged in an easy-to-use phylogeny, which permits the user to select by a single click either an individual species or to select all species within an evolutionary node. For example, clicking the Sophophora subgenus node will result in a search of the genomes of all 18 sequenced members of that subgenus.

FlyBase BLAST offers standard BLAST options (such as TBLASTN) as well as advanced options where users can specify the matrix, word size, codon bias and complexity filters. The BLAST results page displays the regions of similarity across the chromosomes and provides a score and *E*-value for each chromosomal arm. Below this, each individual sequence alignment is shown with their respective scores, *E*-values and sequence information. We would like to highlight here a particularly useful feature of our BLAST output that allows users to directly map BLAST hits onto the *Drosophila melanogaster* genome (note that this feature is also available for the other 11 Drosophilid genomes that are also represented in GBrowse). Every BLAST hit has a button that links to our genome browser, GBrowse. This allows users to visualize the BLAST hit within the context of the surrounding genome, which can be particularly useful when assessing whether a hit is biologically significant or whether to study a candidate gene further.

GBrowse is a Generic Model Organism Database (GMOD, http://gmod.org) tool that displays genomes and their features in a greatly customizable form. Genome features are stretches of sequence that have been either assigned a function (e.g. Insulator Class I sites, exon junctions) or are reported to be the location of an event (e.g. a mutation or transgenic insertion). A group of features of a certain type is called a ‘track’. For example, all exon junctions for all transcripts are grouped into the same track, named ‘RNA-Seq exon junctions’. FlyBase has recently integrated GBrowse version 2, and it has many functions that were not present in the first version of GBrowse. Unlike the previous version, which displayed different views of the genome with fixed tracks, GBrowse 2 allows all tracks to be displayed on the same view at the same time. The tracks are fully customizable; color, shape and even position on the display are all user configurable.

**Figure 3.**
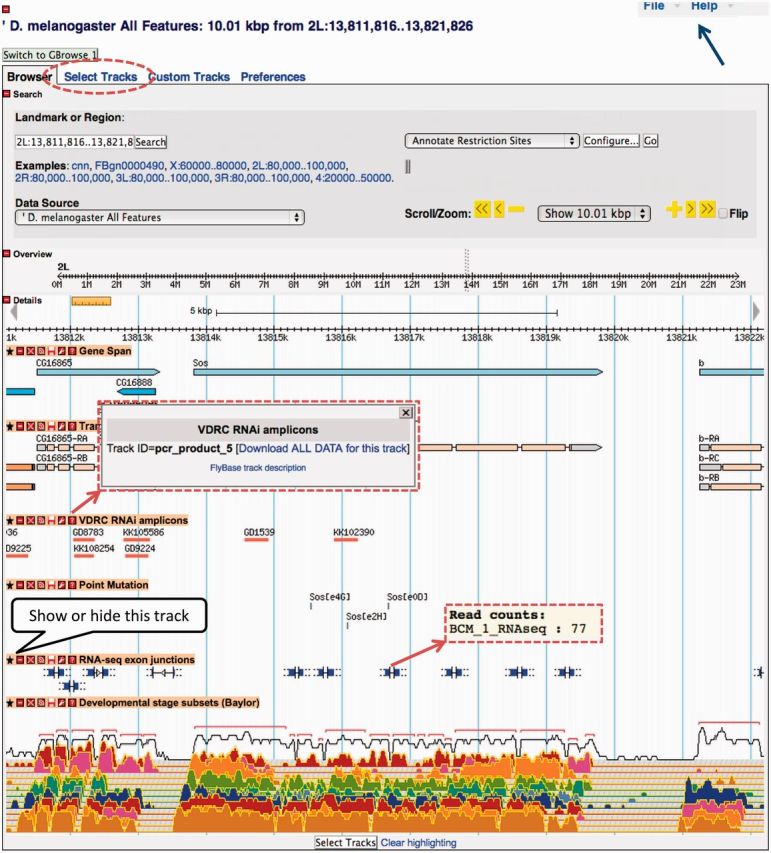
GBrowse—the FlyBase Genome Browser. Tracks are selected by clicking on the ‘Select Tracks’ tab at the top of the browser (highlighted with the red dotted circle), or the ‘Select Tracks’ button at the bottom of the page. The tracks are completely configurable, and can be favorited, opened/closed, shared and even changed in color and appearance. In addition, clicking on a track allows you to order the tracks, so tracks of interest can be placed next to each other. Information on each track can be found by clicking on the ‘?’. Help on how to use GBrowse can be found by clicking on the ‘Help’ button on the top right-hand side (arrow).

Information about feature tracks, including help documentation and links to relevant references, can be found by clicking the ‘?’ icon near the track name in the browser window and then clicking ‘FlyBase track description’ from within the pop-up (Figure 3). Alternatively, on the ‘Select Tracks’ view, there are ‘?’ icons next to each track name which link directly to the documentation (not shown). As for the individual features themselves, in most cases mousing over them evokes a pop-up window containing some summary information about the feature (Figure 3 illustrates this with RNA-Seq exon junction read count pop-up). Clicking on the feature itself will take the user to the report page for that feature, where available. Reciprocally, those report pages concerning features that can be mapped to the genome have links (via the small GBrowse glyphs) directly to the relevant genomic location in GBrowse.

### FeatureMapper

FeatureMapper (http://flybase.org/static_pages/featuremapper/featuremapper.html), our newest query tool, was developed to allow users to search for specific types of genome features in one or more sequence regions. Genome features include functional elements (e.g. gene models, enhancers, protein-binding sites), locations of mutational events (e.g. point mutations, transgene insertion sites) and reagents (e.g. RNAi amplicons or oligo ranges). Previously, access to these types of data using FlyBase tools was restricted to browsing GBrowse or using our pre-computed GFF3 files. This tool allows a user to find the data from GBrowse, but in tabular format and for multiple independent sequences simultaneously.

To use FeatureMapper, you may enter one or more sequence landmarks, and your list can be a mix of types. The only requirement is that each landmark must have a sequence location. For example, you can enter a list of gene symbols (e.g. Kr, nos), transgenic insertion site IDs (e.g. FBti0003342) and sequence ranges (e.g. 3R:14,980,960 … 14,987,924). Next, there is a choice of the region to search. By default, the search is set to the full extent of the landmark and to include features that only partially overlap the landmark. Hence, for a gene, the search would look for all features whose extents overlap any part of the gene span. There are also options to search upstream or downstream of the landmark. One of these latter options is recommended when landmarks are just points along the genome (such as transgenic insertion sites, aberration breakpoints, etc.). The range of sequence to be searched should be entered (in kilobases) for these latter two options.

Once the landmarks are entered, the genome features of interest can be chosen. Genome features are categorized into: gene models (e.g. genes, five_prime_UTR, etc.), aligned evidence (e.g. cDNAs, RNA-Seq exon junctions), noncoding features (e.g. regulatory regions, protein-binding sites), microarray features (Affymetrix v1 and v2), mapped mutations (e.g. point mutations, indels) and RNAi reagents and data (e.g. VDRC RNAi amplicons). There is no limit to the number of features that can be selected (though there may be slower performance if many landmarks are entered and many features are selected).

There are two options for viewing results. Features can be grouped by type (default) or by sequence location (deselect ‘Group output features by type’). Output can be viewed as an HTML table (default) or as GFF lines. Regardless of the input options, the results page shows the list of landmarks in the order in which they were entered. If the default settings are used, there are links to hitlists for the various feature types, so the results of a FeatureMapper search can be exported to Batch Download, QueryBuilder or to a simple text file via the hitlist.

## TARGETED AND COMBINATORIAL QUERYING

Many of our tools are designed specifically to allow searching of one type of data at a time, but in some cases one may need to carry out more complex searches that combine data from multiple data-types across FlyBase. Complex queries of the database can be carried out in two ways: either by using our QueryBuilder search tool or with Structured Query Language (SQL) queries (for those with more knowledge of database structure).

### QueryBuilder

QueryBuilder (http://flybase.org/.bin/qbgui.fr.html) is a sophisticated web-based search tool that allows combinatorial searching of any fields from any report in FlyBase. Its advanced search capability takes maximum advantage of the data field layout in the underlying Chado database, but its easy-to-use interface means that absolutely no knowledge of this table structure is required. This tool is available both from our home page icon bar as well as from the Tools drop-down menu.

Query segments are built one by one to create complex searches. Data fields are chosen that are specific to a given data type (e.g. the Symbol field from the Gene Report, or the Author field from the Reference Report). After the data type of interest is selected, only appropriate fields are shown as options for each data type, and the fields are presented in similar order and format as in FlyBase report pages to aid navigation. Individual query segments can then be combined using Boolean operators (AND, OR and BUT NOT) to build up complex searches. Depending on the fields selected, search criteria can include text strings, CV terms or numbers. Number fields can include calculations and logical functions such as greater than (>) or less than (<), and for many fields an index dictionary is available to allow you to see the most commonly used terms. Wild cards are also allowed, to give you the best chance of carrying out the most appropriate search. An auto-complete function facilitates entry of productive queries.

QueryBuilder allows for an almost infinite number of data combinations, and we appreciate that this can be quite daunting when you first come to use it. To help you get started, and to act as an educational tool, we have created a series of pre-constructed templates that cover the most commonly used QueryBuilder queries. When you click on the QueryBuilder button on the homepage you are presented with three options: ‘Use a query template’, ‘Import a saved query’ or ‘Build a new query’. For first time users we recommend the ‘Use a query template’ option as a starting point. These templates are divided into sections according to data type and are fully editable allowing you to adapt them to your individual search criteria.

#### Example

As an example, let’s say we are interested in obtaining stocks that have wing vein mutant phenotypes, but we do not want those that also have wing margin defects. To do this, we need to identify alleles with wing vein phenotypes that have available stocks, but remove those that are also annotated as having a wing margin defect. This kind of multi-leg query is beyond the capabilities of QuickSearch or TermLink, but is straightforward in QueryBuilder.

**Figure 4.**
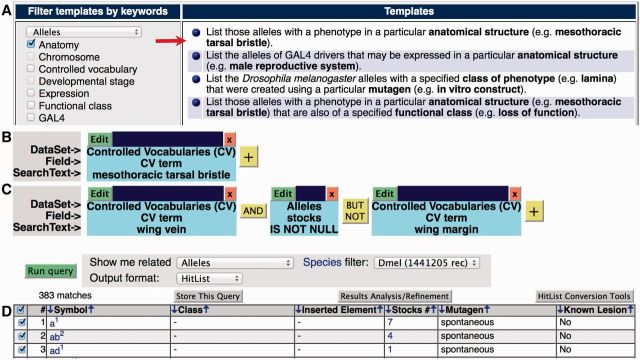
QueryBuilder. **(A)** Available templates for alleles and anatomy. The template used in the example is indicated with an arrow. (**B**) The template query segments. (**C**) The template query segments edited for the example query. (**D**) The resulting hitlist. In total, 383 alleles match the search criteria.

For the example search we are interested in allele phenotypes, so we need to look at the available ‘Anatomy’ queries in the ‘Alleles’ section of the Templates, and choose the query that is most similar to the search we would like to carry out. In this case, the first option works the best: ‘List those alleles with a phenotype in a particular anatomical structure (e.g. mesothoracic tarsal bristle)’ (Figure 4A). On choosing this option, the query segments required to carry out this search are displayed (Figure 4B), and we are able to make changes to the search. For this example, we need to alter the search criteria in the first segment to use the CV term we are interested in, i.e ‘wing vein’, by clicking the green Edit box. To ensure we get the correct CV term we need to search in the ‘Controlled Vocabulary (CV)’ DataSet for ‘wing vein’ and insert the matching term, in this case ‘wing vein; FBbt:00004751’. We then need to click the yellow ‘+’ box to add two additional segments; one to limit the search to alleles that have stocks, and one to exclude all alleles that are annotated with the anatomy CV term ‘wing margin’. The resulting query segments are illustrated in Figure 4C. Various output options are available on QueryBuilder, and the results are presented in a hitlist underneath the query. A total of 383 alleles match our search criteria, using fb_2013_05, but you will get a different number using a more recent FlyBase update (Figure 4D).

If you are likely to rerun the same query, you can save the search as a text file (by clicking on ‘Store this Query’), and then rerun it by using the ‘Import a saved query’ option. For those who are already familiar with QueryBuilder or who are unable to find a template that matches their search criteria, the ‘Build a new query’ option allows you to create all of the query segments from scratch.

In addition to searching the whole database, you are also able to upload a pre-defined list of entities, for example, a list of genes, to use as a dataset to search. This can be achieved either by uploading an external list from a file, or by transferring lists from other tools using the HitList Conversion Tools button, as described for the Termlink and RNA-Seq searches in the previous sections. Simply choose ‘QueryBuilder (new session)’ from the HitList Conversion Tools options to make the list a segment of a new QueryBuilder query.

### Querying our pre-computed files or by using SQL

In addition to the web-based search tools on FlyBase, such as QuickSearch and QueryBuilder, FlyBase provides access to all our data through a series of pre-computed data files as well as direct access to a public read-only instance of the database that can be queried through SQL.

Our pre-computed files can be found in the Files menu. This menu provides a number of options, including access to our FTP site (ftp://ftp.flybase.net/). At the FTP site, various cuts of the data are provided, ranging from GFF and FastA files to Chado XML data sliced into portions such as chromosomes, genes, intergenic regions and syntenic regions. These data are provided for the current genome release and all older releases for the 12 sequenced *Drosophila* genomes. The latest, most up-to-date *D. melanogaster* genome can be found in the Drosophila_melanogaster-current subdirectory.

In addition to our FTP site, we provide pre-computed cuts of the data for download direct from the FlyBase website. A description of the available data can be found in the Files Overview help document at the top of the Files menu (http://flybase.org/static_pages/docs/datafiles.html). In brief, data relating to the current release of FlyBase data can be accessed via the ‘Current release’ option, with selected previous releases giving an overview of the data back to November 2004. Pre-computed downloads are available for many of the objects found in FlyBase, such as genes (e.g. Gene—GO association data), alleles (e.g. XML versions of all the data found on allele report pages) and stocks (e.g. a tab-separated file of all the stock records in FlyBase). In addition, we provide downloads of every CV used in FlyBase, tables of FlyBase references with their PubMed identifiers and tables of genomic coordinates with cytological location. If there is a slice of the data that you would like but you cannot find on our pre-computed file list, please email us and we will try to assist you.

Alternatively, if you are familiar with SQL, we allow direct searching of our Chado schema PSQL database (9). Direct access for moderate usage requires a PostgreSQL client application installed on the user’s computer (see www.postgresql.org/ for PostgreSQL software and documentation). For extensive usage, we recommend that a copy of the FlyBase PostgreSQL Chado database be installed locally. The PostgreSQL dumps can be downloaded from ftp://ftp.flybase.net/releases/current/psql. See the README file for further information (ftp://ftp.flybase.net/releases/current/psql/README).

Like any relational database, information in FlyBase is stored in fields that are arranged in tables. Understanding the relationship between these tables is key to the formulation of SQL to query the data. Example SQL queries that can be used to learn about the structure of the database can be found on the FlyBase forum (http://flybase.org/forums/viewforum.php?f=14). In addition, documentation, including the FlyBase field mapping tables, is available on the GMOD website (http://gmod.org/wiki/FlyBase_Field_Mapping_Tables).

## CONCLUSIONS

For over 20 years, FlyBase has provided a portal into the world of *Drosophila* genes and genomes. As the volume and complexity of published fly-related data have grown over the years, so too has FlyBase. With manually curated data from over 42 000 references, and data on over 122 000 fly stocks, along with large-scale genomic data incorporated from recent ‘big data’ initiatives, FlyBase has evolved into a comprehensive and complex database. In this article, we have illustrated some of the more advanced methods with which you can query these data, such as using QueryBuilder for complex combinatorial searches, using SQL to search the database tables behind FlyBase, and using BLAST, GBrowse and our new FeatureMapper tool to explore genomic data. The addition of new types of data can sometimes necessitate the development of new tools, and this has certainly been the case for the RNA-Seq data and the development of the RNA-Seq Search tool. Over the coming years, we expect FlyBase to continue to incorporate new datasets and to continue to develop new ways to display and query these data.

We welcome user feedback on any aspect of FlyBase, and we encourage interaction through our ‘Contact FlyBase’ form, accessible from the footer found on every page. We suggest FlyBase be referenced in publications by citing this publication and the FlyBase URL (http://flybase.org).

## FUNDING

National Human Genome Research Institute at the National Institutes of Health [P41 HG00739]; Medical Research Council (UK) [G1000968]. Funding for open access charge: NIH NHGRI.

*Conflict of interest statement*. None declared.
